# Predicting longevity-related traits in Swiss low-input and organic dairy cows from herdbook data of first versus second lactation

**DOI:** 10.1016/j.vas.2026.100707

**Published:** 2026-05-19

**Authors:** Anna Bieber, Dirk Hinrichs, Florian N. Moser, Ariane Maeschli, Isabella Lora, Giulio Cozzi, Florian Leiber

**Affiliations:** a Department of Livestock Sciences, Research Institute of Organic Agriculture FiBL, Ackerstrasse 113, 5070 Frick, Switzerland; b Department of Animal Breeding, University of Kassel, Nordbahnhofstr. 1a, 37213 Witzenhausen, Germany; c Department of Animal Medicine, Production and Health MAPS, University of Padova, Viale dell'Università 16, 35020 Legnaro (PD), Italy

**Keywords:** Productive lifespan, Robustness, Resilience, Survival, Test-day records

## Abstract

Dairy cows’ longevity integrates multiple functional traits and influences dairy production sustainability. This study assessed the impact of productive lifespan on lifetime productivity in Swiss low-input and organic dairy farms, compared the predictability of longevity and lifetime production using first- versus second-lactation data, and evaluated whether lactation curve parameters (LCP) derived from test-day records improve predictions. Herdbook data of culled cows were analyzed using mixed-effects models to predict length of productive lifespan (LPL; days from first calving to culling), number of lactations until culling (MaxLN), lifetime milk production (LTP; kg energy-corrected milk, ECM), and average daily milk yield during productive lifespan (DMY_LPL; kg ECM). Records from first and second lactation were used in three datasets: a large dataset (n = 10,031 cows, 384 farms), a dataset with insemination records (n = 6,011 cows in first and 5,662 in second lactation, 372 farms), and a dataset with LCP (n = 6,048 cows in first and 6,735 in second lactation, 384 farms). Models based on second-lactation data with insemination records performed best for LPL and LTP, whereas MaxLN was best predicted using first-lactation data with insemination records. Predictability remained low for LPL (0.7%) and MaxLN (0.8%), and moderate for LTP (10.0%). Models including LCP best predicted DMY_LPL (64.9%). Predictive performance improved with additional factors, including somatic cell count, breed, calving interval, age at first calving, lactation persistency, milk yield traits, fat-to-protein ratio, and alpine pasturing. Based on second-lactation data, lifetime milk yield traits were reasonably predictable, whereas longevity prediction remained challenging.

## Highlights

• Second-lactation data predict lifetime production better than first-lactation data.
• Insemination records improve model fit but longevity predictability stays low.
• Lactation curve parameters yield moderate accuracy for lifetime daily production.
• Lifetime production is predictable; longevity remains challenging to forecast.

## 1 Introduction

Extending dairy cow longevity is essential for improving the economic, environmental, and ethical sustainability of dairy production systems. Economically, a longer productive lifespan allows rearing costs to be distributed over more years, enhancing profitability (Essl, 1998; Bergeå et al., 2016). Moreover, peak annual milk yield is typically reached in the fifth lactation, with the highest annual profit occurring in the sixth lactation in organic dairy cows (Horn et al., 2012). However, the optimal economic lifespan remains uncertain and likely varies by breed and production system (De Vries and Marcondes, 2020).

From an environmental perspective, extending productive lifespan improves efficiency by spreading resource use and greenhouse gas emissions over a longer production period, as cows kept longer reduce the number of replacement heifers and dilute greenhouse gas emissions associated with rearing (Essl, 1998; van Middelaar et al., 2014; Adamie et al., 2023). Additionally, methane emissions have been reported to decrease after dairy cows reach 6.5 years of age under experimental conditions (Grandl et al., 2016). Finally, the link between longevity and animal health and welfare underscores the ethical importance of promoting longevity as a desirable goal (Oltenacu and Broom, 2010; Ahlman et al., 2011). Comparative studies of farms with contrasting longevity profiles showed that longer productive lifespan was associated with a higher proportion of loose housing systems (Eppenstein et al., 2023) and better fertility (Bieber et al., 2024).

Despite these benefits, productive lifespan has declined in most dairy systems (Schuster et al., 2020; Dallago et al., 2021), for example in US Holstein dairy cows since the 1960s (Oltenacu and Broom, 2010). Currently, productive lifespan averages 2.5–4 years, with total lifespan from birth to death ranging from 4.5–6 years (De Vries and Marcondes, 2020). In Switzerland, organic dairy cows have a productive lifespan of 3.5–4.3 lactations (Bieber et al., 2019), while the national average is 3.7 lactations (Burren, 2011). This is longer than the 2.9–3.1 lactations in Swedish dairy cows and 2.5 lactations in German organic herds under higher production intensities (Bieber et al., 2020). Nevertheless, current longevity remains far below the estimated physiological potential of 20 years (Hoffman and Valencak, 2020).

The primary culling reasons are involuntary culling events due to health, fertility, or conformation problems (Pinedo et al., 2010; Ahlman et al., 2011; Pritchard et al., 2013a; Olechnowicz et al., 2016; Schuster et al., 2020). Additionally, external factors such as the availability of replacement heifers and economic factors (e.g. milk prices) play a significant role in determining longevity (Alvåsen et al., 2018; De Vries and Marcondes, 2020). Consequently, improving longevity has become a key focus in dairy breeding and management (De Vries and Marcondes, 2020), particularly in organic dairy production systems (Horn et al., 2012). However, successful selection for longevity may slow genetic progress in other desirable traits due to the extended generation intervals (Stefani et al., 2018).

A practical approach to improving longevity is the use of easily accessible herdbook data to estimate lifetime performance. Key herdbook traits that can be utilized include age at first calving (Eastham et al., 2018; Sawa et al., 2019), calving interval (Nor et al., 2014), lactation curve parameters (Poppe et al., 2020), somatic cell count (Archer et al., 2013), and breed (Bieber et al., 2019; Bieber et al., 2020).

From a practical perspective, culling decisions are made throughout early productive life, with high culling rates reported for early lactations (De Vries et al., 2010; Horn et al., 2012; Olechnowicz et al., 2016). This highlights the need for reliable early-life predictors of longevity and lifetime performance based on routinely available herdbook data.

The objective of this study was to develop predictive models using herdbook data from first versus second lactation to estimate longevity and lifetime production in Swiss dairy cows. Specifically, we aimed to answer the following key questions:

(i) How does productive lifespan affect productivity in Swiss low-input dairy systems?
(ii) Does predictive performance for longevity-related traits differ between models trained on first- versus second-lactation data?
(iii) Do lactation curve parameters derived from test-day records improve longevity predictions?

We hypothesized that prediction accuracy would be higher using second-lactation data and that inclusion of lactation curve parameters would improve predictions.

## 2 Materials and methods

### 2.1 Data collection, trait definitions and trait calculation

Data for this study were extracted from the Oracle data repository of Qualitas AG (Zug, Switzerland), which is the competence center for informatics and genetics of Swiss breeding associations. The repository contained information on 580 low-input and organic dairy farms involved in research projects or extension activities of the Research Institute of Organic Agriculture, FiBL (https://fibl.qualitasag.ch/FiBL/, accessed 23.04.2020). For this study herdbook data of cows registered with the “Braunvieh Schweiz” or “swissherdbook” breeding associations were selected from farms with at least nine cows in second lactation between 1 January 2008 and 31 December 2012. Only cows with recorded culling date were included, and the culling dates for these cows ranged from 26 August 2008 to 20 April 2020. Cows missing data on milk yield, milk composition traits, somatic cell count, or those that changed farm were excluded. The extracted variables included date of birth, age at first calving (AFC, months), breed, lifetime milk production and parity at culling. Herd size was estimated by dividing the total number of test-day records per farm and year by nine, based on the assumption of an average of nine test-day records per 305-day lactation (Ivemeyer et al., 2009). Herd size was then assigned to cows according to their calving year. The following variables were extracted for both the first and second lactation:

• lactation persistency = (cumulative milk yield (kg) from days in milk (DIM) 101–200 / cumulative milk yield (kg) from DIM 1–100),
• test-day records on milk yield (kg), protein content (%), fat content (%), and somatic cell count (cells/mL of milk),
• alpine pasturing: binary variable (0 = no, 1 = yes) based on the information whether a cow grazed in alpine areas during the respective lactation,
• calving season: binary variable with “winter” (September to February) and “summer” (March to August),
• calving year: running from 2005 to 2012 (for first lactations) and from 2008 to 2012 (for second lactations).

Additional traits were the average fat-to-protein ratio (FPR = fat content (%) / protein content (%)) and the proportion of test-day records with somatic cell counts exceeding 100,000 cells/mL (SCC100 = (Number of test-day records with SCC > 100,000 cells/mL / Total number of test-day records) × 100) as udder health indicator.

### 2.2 Target trait definition

The study considered the following longevity and productivity traits (summarized in Table 1):

• Length of productive lifespan (LPL): number of days from first calving to culling.
• Maximum number of lactations achieved until culling (MaxLN): total number of calving events until culling.
• Lifetime milk production (LTP, ECM kg): total milk yield corrected for fat (4.0%) and protein (3.4%) using the equation [1] (Heller and Potthast, 1990):
  (1) LTP(kgECM)=Milk(kg)×[0.38×Fat(%)+0.21×Protein(%)+1.05]/3.28,
• Daily milk yield during length of productive lifespan (DMY_LPL, kg ECM): calculated as LTP (kg ECM) divided by LPL (days).

**Table 1:** Overview on abbreviations and definitions of target traits in this study.

Trait	Abbreviation	Definition
Length of productive lifespan (days)	LPL	Number of days from first calving until culling
Number of lactations achieved	MaxLN	Total number of calving events until culling
Lifetime milk production (kg ECM)	LTP	Lifetime total milk production in kg energy-corrected milk
Daily milk yield during length of productive lifespan (kg ECM)	DMY_LPL	Average daily milk yield during productive lifespan in kg energy-corrected milk (LTP / LPL)
Daily milk yield during length of total life (kg ECM)	DMY_LT	Average daily milk yield during total life in kg energy-corrected milk (LTP / days from birth to culling)

Spearman’s rank correlation coefficients (rho) between the number of lactations achieved and lifetime performance traits were estimated using the “cor” function in R to assess the relationship between longevity and productivity.

### 2.3 Lactation curve parameter estimation

Milk yield (kg) from all available test-day records of cows with at least six test-day records was used to estimate lactation curves. Lactation curves were fitted separately for first and second lactation using the incomplete gamma function model of Wood (1967) (equation [2]) implemented in the “easyreg” R package (version 4.0; Arnhold, 2018) in R (version 3.6.3; R Core Team, 2020):

(2) $$DMY=at^{b}exp\left(-ct\right),$$

where DMY is daily milk yield (kg), *a, b* and *c* are Wood model coefficients, and t is the day in milk of the respective test-day record. Coefficient *a* reflects the initial production level and determines the overall height of the lactation curve; cows with higher *a* estimates start lactation at higher production level. Coefficient *b* describes the ascending phase toward peak production, with larger values indicating a steeper rise. Coefficient *c* represents the rate of decline after the lactation peak; smaller values indicate greater lactation persistency, whereas larger values correspond to a faster decline in milk yield (Bouallegue and M’Hamdi, 2019).

**Fig. 1:**
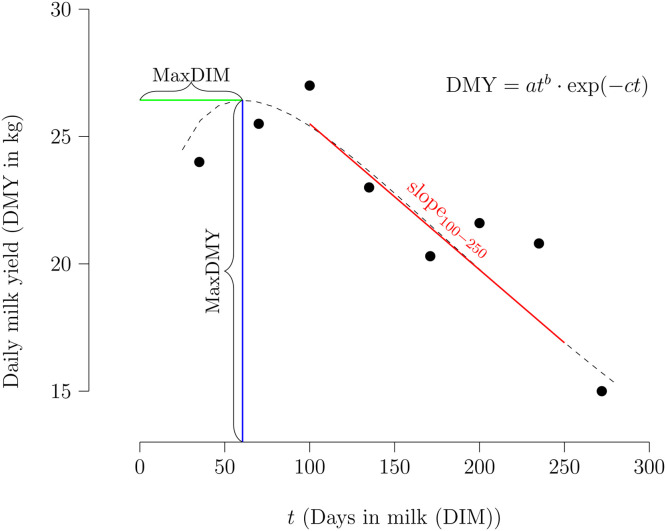
Lactation curve parameter estimation (Wood, 1967).

The lactation curve model is illustrated in Fig. 1. Lactation curve parameters (LCPs) were estimated per cow and lactation using the test-day records on milk yield and days in milk. Derived lactation curve parameters were calculated from the fitted Wood coefficients (*a, b, c*) and included the maximum daily milk yield (MaxDMY), the day of maximum daily milk yield (MaxDIM), and the slope of milk yield between days in milk 100 and 250 (Slope_100-250_). Additionally, the model’s coefficient of determination (R^2^) was calculated.

Footnote: Example of the incomplete gamma model to estimate the lactation curve (broken line) from test-day records (black dots) at lactation level using the formula of Wood (1967) as indicated on the upper right. From each lactation curve we extracted the model's goodness of fit (R2), and the following lactation curve parameters: the maximum daily milk yield (MaxDMY, blue line, here 26 kg), the day in milk at which the maximum daily milk yield is reached (MaxDIM, green line, here 60 days), and the slope of the milk yield between days in milk 100 and 250 (Slope100-250, brown line).

### 2.4 Data selection and resulting datasets

The initial dataset comprised 10,312 dairy cows from 388 farms. Records were filtered using the following criteria: exclusion of breeds with fewer than 20 cows and herds with fewer than 9 animals; lactation number at culling between 2 and 20; lactation length between 200 and 720 days in milk (DIM); lactation persistency greater than zero; and age at first calving between 20 and 42 months. For second lactation analyses an additional restriction on calving interval (260–750 days) was applied. After filtering, the resulting dataset, hereafter referred to as dataset_big_, contained 10,031 cows from 384 dairy farms.

Dataset_big_ was further filtered to retain only cows with recorded data on number of inseminations in the respective lactation. The resulting dataset (dataset_ins_) comprised 6,011 cows for first lactation and 5,662 cows for second lactation from 372 farms. The reduced size of dataset_ins_ reflects the restriction to cows with complete insemination records.

Additionally, datasets including lactation curve parameters (LCPs) derived from test-day records in first (LN1) and second lactation (LN2) were generated. Curve estimation was successful for 9,423 cows (LN1) and 10,003 cows (LN2) from 384 farms. The estimated Wood model parameters were:

LN1: *a* = 19.88 ± 0.14 SE, *b* = 0.083 ± 0.002 SE, and *c* = 0.0025 ± 0.00002 SE;
LN2: *a* = 25.94 ± 0.17 SE, *b* = 0.069 ± 0.002 SE, and *c* = 0.0031 ± 0.00002 SE,
indicating high precision of model estimation.

To ensure numerical reliability and biological plausibility, estimated lactation curves were subjected to a multi-criteria validation procedure, as follows.

First, curves with poor goodness-of-fit (R^2^ < 0.5) were excluded.

Wood model coefficients were required to meet physiological constraints (ascending phase coefficient *b* > 0; declining phase coefficient *c* > 0). Coefficient estimates violating these conditions were considered indicative of non-physiological lactation shapes (e.g. linear or U-shaped curves) and were excluded.

Third, implausible peak characteristics (MaxDMY < 5 or > 80 kg/day or MaxDIM < 1 or > 120 DIM) were removed.

Finally, outliers in Wood model coefficients (*a, b*, and *c*) and derived MaxDMY were identified using a robust, non-parametric criterion based on the interquartile range (IQR) calculated across all fitted lactation curves within each lactation. Coefficient estimates (*a, b, c*) or derived MaxDMY values below the first quartile minus three times the IQR or above the third quartile plus three times the IQR were classified as extreme. Lactation curves exhibiting at least one such extreme value were considered numerically unreliable and excluded.

Only lactation curves fulfilling all validation criteria were retained in two final validated datasets (dataset_LCP_) comprising 6,048 cows (LN1) and 6,735 cows (LN2) from 384 farms. Details on the validation steps and data reduction are provided in Supplementary Table S1.

An overview of validation criteria and resulting datasets is shown in Table 2. Herd-level descriptive statistics are shown in Table 3 for dataset_big_. Lactation-level summaries are provided in Table 4a for dataset_big_, Table 4b for dataset_ins_, and Table 4c for dataset_LCP_. Breed distributions across datasets are provided in Supplementary Table S2.

**Table 2:** Overview of data validation criteria and resulting datasets.

Dataset	N cows		N farms	description	Data inclusion criteria
	LN1	LN2			
dataset_initial_	10,312	10,312	388	Initial dataset extracted from the Oracle data base	- Farms with ≥ 9 cows with second lactation test-day data between 1.1.2006 and 1.1.2013 - Members of the Braunvieh CH or swissherdbook breeding association
dataset_big_	10,031	10,031	384	Validated dataset	- breeds with > 20 cows - herds with ≥ 9 cows - lactation length between 200 and 720 days in milk - lactation persistency^1^ of > 0, - age at first calving between 20 and 42 months. - For second lactation, a calving interval between 260 and 750 days
dataset_ins_	6,011	5,662	372	Subset of data_big_ selected for data on number of inseminations	- Presence of data on number of inseminations
dataset_LCP_	6,048	6,735	384	Dataset containing validated lactation curve parameter estimates for first and second lactation	- Data on LCP^2^ derived from Wood model and validated according to the following criteria: - R^2^ ≥ 0.5 - Wood model coefficients *b* (ascending phase) > 0, and *c* (declining phase) ≥ 0 - MaxDMY ≥ 5 and ≤ 80 kg milk - MaxDIM ≥ 1 and ≤ 120 days in milk - Outlier exclusion^3^ for *a, b, c*, and MaxDMY for estimates outside ± 3 × IQR

LN1 = first lactation, LN2 = second lactation

1 **Lactation persistency:** (cumulative milk yield (kg) from days in milk (DIM) 101–200 / cumulative milk yield (kg) from DIM 1–100).

2 **Lactation curve parameters (LCP)** were derived from the Wood model. The model coefficients *a, b*, and *c* represent the scale, ascending phase, and declining phase of the lactation curve, respectively. These coefficients were used to calculate derived lactation curve parameters, including peak daily milk yield (MaxDMY, kg), day in milk at peak yield (MaxDIM), and slope between DIM 100 and 250 (Slope_100–250_). R² denotes the coefficient of determination of the Wood model fit.

3 **Outlier detection:** based on a robust interquartile range (IQR) criterion. Within lactation, quartiles Q1 (25th percentile) and Q3 (75th percentile) were calculated for each trait, with IQR = Q3 - Q1. Values < Q1 - 3 × IQR or > Q3 + 3 × IQR were classified as extreme and excluded.

**Table 3:** Summary statistics of dataset_big_ at herd level.

	dataset_big_ N cows = 10,031, farms = 384		
Trait^1^	Mean ± SD	Median	Min – Max
Herd size (cows)	32.1 ± 17.4	28.1	10–89
LPL (days)	1,689 ± 776	1,569	508–4,509
MaxLN (lactations)	4.7 ± 2.0	4.0	2–11
LTP (kg ECM)	33,870 ± 17,068	30,737	4,700–108,310
DMY_LPL (kg ECM)	19.9 ± 3.7	19.7	5.3–38.5
AFC (months)	29.6 ± 3.9	29.0	20–42
CI_1_ (days)	391 ± 62	373	262–748

SD = standard deviation, Min = minimum, Max = maximum,

1 Trait: LPL = length of productive lifespan in days, MaxLN = number of lactations until culling, LTP = lifetime milk production in kg energy-corrected milk (ECM), DMY_LPL = average daily milk yield during length of productive lifespan in kg ECM, AFC = age at first calving in months, CI_1_ = first calving interval in days

**Table 4a:** Descriptive statistics of first and second lactation records in dataset_big_.

	First lactation N cows = 10,031, farms = 384			Second lactation N cows = 10,031, farms = 384		
Trait^1^	Mean ± SD	Median	Min - Max	Mean ± SD	Median	Min - Max
ECM (kg)	6,542 ± 1651	6,330	1,429–17,468	7,383 ± 1,943	7,135	1,561–21,638
DIM	329 ± 58	315	212–692	327 ± 60	313	203–718
Fat (%)	4.06 ± 0.43	4.02	2.51–7.01	4.08 ± 0.46	4.04	2.51–7.40
Protein (%)	3.35 ± 0.23	3.34	2.48–5.06	3.41 ± 0.26	3.39	2.60–4.91
FPR	1.21 ± 0.11	1.20	0.80–1.90	1.19 ± 0.12	1.20	0.80–1.90
SCC100 (%)	23.9 ± 28.6	11.1	0–100	34.5 ± 30.6	28.6	0–100
PERS	86.4 ± 10.0	86.0	41–153	80.8 ± 9.8	81.0	34–136

SD = standard deviation, Min = minimum, Max = maximum

1 Trait: ECM = energy-corrected milk yield in kg, DIM = days in milk, FPR = fat-to-protein ratio, calculated as fat content (%) / protein content (%), Fat = fat content in %, Protein = protein content in %, SCC100 = proportion of test-day records with a somatic cell count of > 100,000 cells/mL milk, PERS = lactation persistency, i.e. cumulative milk yield (kg) from days in milk (DIM) 101–200 divided by cumulative milk yield (kg) from DIM 1–100.

**Table 4b:** Descriptive statistics of first and second lactation records in dataset_insem_.

	First lactation N cows = 6,011, farms = 372			Second lactation N cows = 5,662, farms = 372		
Trait^1^	Mean ± SD	Median	Min – Max	Mean ± SD	Median	Min – Max
ECM (kg)	6,629 ± 1,717	6,378	1,429–17,468	7,375 ±1,984	7,089	1,790–21,638
DIM	335 ± 63	319	212–692	330 ± 63	314	217–698
Fat (%)	4.09 ± 0.42	4.04	2.51–7.01	4.11 ± 0.47	4.05	2.51–7.40
Protein (%)	3.39 ± 0.24	3.38	2.48–4.49	3.45 ± 0.26	3.44	2.62–4.71
FPR	1.21 ± 0.11	1.20	0.80–1.90	1.19 ± 0.12	1.20	0.80–1.90
SCC100 (%)	23.4 ± 27.8	11.1	0–100	36.4 ± 30.5	30.0	0–100
PERS	86.2 ± 9.8	86.0	41–153	80.7 ± 9.7	81.0	34–129
INS	1.83 ± 1.16	1.00	1–9	1.85 ± 1.25	1.00	1–9

SD = standard deviation, Min = minimum, Max = maximum

1 Trait: ECM= energy-corrected milk yield in kg, DIM = days in milk, FPR = fat-to-protein ratio, calculated as fat content (%) / protein content (%), Fat = milk fat content in %, Protein = milk protein content in %, SCC100 = proportion of test-day records with a somatic cell count of > 100,000 cells/mL milk, PERS = lactation persistency, i.e. cumulative milk yield (kg) from days in milk (DIM) 101–200 divided by cumulative milk yield (kg) from DIM 1–100, INS = number of inseminations

**Table 4c:** Descriptive statistics of first and second lactation records and test-day recording properties in datasets with lactation curve parameters (LCPs).

	First lactation N cows = 6,048, farms = 384			Second lactation N cows = 6,735 farms = 384		
Trait^1^	Mean ± SD	Median	Min - Max	Mean ± SD	Median	Min - Max
ECM (kg)	6,565 ± 1,627	6,357	1,331–17,457	7,369 ± 1,894	7,165	1,478–20,962
DIM of lactation	328 ± 56	315	212–692	323 ± 55	311	203–718
Fat (%)	4.04 ± 0.43	4.00	2.60–7.01	4.06 ± 0.46	4.01	2.53–7.40
Protein (%)	3.35 ± 0.23	3.33	2.60–5.06	3.40 ± 0.25	3.38	2.62–4.80
FPR	1.21 ± 0.11	1.20	0.80–1.90	1.20 ± 0.12	1.20	0.80–1.90
SCC100 (%)	24.1 ± 28.5	12.5	0–100	34.2 ± 29.9	28.6	0–100
MaxDMY	25.7 ± 4.6	25.4	11.8–48.0	31.1 ± 6.4	30.8	8.8–61.6
MaxDIM	46.2 ± 21.8	45.0	5–120	36.9 ± 19.7	36.0	1–119
Slope_100-250_	-0.05 ± 0.02	-0.05	-0.16– -0.01	-0.07 ± 0.03	-0.07	-0.20– -0.01
N test-day records	8.75 ± 1.75	9	6–10	8.97 ± 0.95	9	7–10
Interval between test-day records (days)	34.2 ± 5.6	34	29–143	34.2 ± 4.8	34	30–207
Days in milk at test-day record	160 ± 91	158	1–437	158 ± 91	156	1–442

SD = standard deviation, Min = minimum, Max = maximum

1 Trait: ECM = energy-corrected milk yield in kg, DIM = days in milk of the whole lactation, FPR = fat-to-protein ratio, calculated as fat content (%) / protein content (%), Fat = milk fat content in %, Protein = milk protein content in %, SCC100 = proportion of test-day records with a somatic cell count of > 100,000 cells/mL milk, PERS = lactation persistency, i.e. cumulative milk yield (kg) from days in milk (DIM) 101–200 divided by cumulative milk yield (kg) from DIM 1–100.

### 2.5 Statistical models

Linear mixed effects models were applied to analyze length of productive lifespan (LPL, days), lifetime milk production (LTP, kg ECM) and average daily milk yield during length of productive lifespan (DMY_LPL, kg ECM). A generalized linear mixed model with a Poisson distribution and log link function was used for maximum lactation number achieved (MaxLN). All models were fitted using the “lme4” package (version 1.1-35.1; Bates et al., 2015) in R (version 3.6.3; R Core Team, 2020).

Prior to model fitting, all explanatory variables were classified as numeric or categorical. Pairwise Pearson correlations among numeric predictors were evaluated, when |r| ≥ 0.7, only one variable per correlated pair was retained based on the findCorrelation procedure from the “caret” package (version 7.0-1; Kuhn, 2008).

In addition, variance inflation factors (VIFs) were calculated for the retained predictors, using the “car” package (version 3.0-6, Fox and Weisberg, 2019). All VIF values were < 3, indicating low multicollinearity.

Analyses were conducted separately for first and second lactation using three dataset-specific models.

a) Model 1 for dataset_big_ (10,031 cows, 384 farms), including routinely available herdbook data:

Y_big_ = μ + AFC + nHerd + Breed + Year + se + Alp + Pers + FPR + SCC100 + MY + (1|Farm) + e_big_ [M1],

b) Model 2 extended Model 1 by incorporating the number of inseminations (INS) and was fitted to dataset_ins_ (LN1: 6,011 cows, LN2: 5,662 cows, 372 farms):

Y_ins_ = μ + AFC + nHerd + Breed + Year + se + Alp + Pers + FPR + SCC100 + MY + INS + (1|Farm) + e_ins_ [M2],

c) Model 3
  $$\begin{array}{c} Y_{\text{LCP}}=\mu +\text{AFC}+\text{nHerd}+\text{Breed}+\text{Year}+\text{se}+\text{Alp}+\text{FPR}\\ +\text{SCC}100+\text{MaxDMY}+\text{MaxDIM}+\text{Slop}e_{100-250}+\left(1|\text{Farm}\right)+e_{\text{LCP}}\left[M3\right], \end{array}$$
  was fitted for dataset_LCP_ (LN1: 6,048 cows, LN2: 6,735 cows, 384 farms) and replaced milk yield and persistency with lactation curve characteristics, allowing a more detailed description of milk production dynamics.

In all models Y denotes the respective response variable and μ the intercept.

Continuous covariates included age at first calving (AFC, months), herd size (nHerd), lactation persistency (Pers; ratio of cumulative milk yield (kg) from DIM 101–200 to cumulative milk yield (kg) from DIM 1–100), fat-to-protein ratio (FPR; fat content (%) / protein content (%)), proportion of test-day records with a somatic cell count of > 100,000 cells/mL milk (SCC100), milk yield (MY, kg ECM), estimated maximum daily milk yield of the lactation curve (MaxDMY, kg), estimated day in milk at peak yield according to the lactation curve (MaxDIM), and the estimated slope of the lactation curve between DIM 100 and 250 (Slope_100-250_).

Fixed effects were breed (Breed; Original Braunvieh, Brown Swiss, Simmental, Swiss Fleckvieh, Montbéliarde, Jersey, Holstein), year of calving (Year; 2006–2012), calving season (se; winter or summer), and alpine pasture (Alp; yes or no).

Random effects included a random intercept for farm (1|Farm) and e _big or ins or LCP_ represents the residual error.

For second-lactation analyses, Models M1–M3 were extended by including the calving interval (CI; days) as an additional covariate, resulting in six models per dependent trait.

Continuous covariates were centered and standardized (mean = 0, SD = 1) to improve numerical stability and facilitate comparison of effect sizes. Categorical variables were specified as factors. A random intercept for farm accounted for clustering.

Model intercepts represent the expected value for an animal in the reference categories (e.g. no alpine grazing, reference breed, summer calving season) at the mean of all continuous covariates. Linear mixed-model estimates are reported as regression coefficients (± standard errors). For MaxLN, results are presented as incidence rate ratios (IRR) with 95% confidence intervals. Wald tests were used to derive P-values for all fixed effects.

For LPL, functional longevity is reported (Schuster et al., 2020), defined as the ability to avoid involuntary culling for reasons other than production.

### 2.6 Model performance and benchmarking

Model performance was evaluated using 10-fold cross-validation repeated five times, with folds generated at farm level. In each iteration, approximately 90% of farms (and all cows within those farms) were used for model training and the remaining 10% for testing, ensuring no farm contributed to both sets and preventing information leakage.

Regression coefficients were estimated on training data only. Predictions were subsequently generated for cows belonging to farms not included in the training set. Consequently, predictions for test farms relied exclusively on the fixed-effects component of the model, without using farm-specific random effects estimates derived from the test data. Each fold served once as test set per repetition, resulting in a total of 50 train-test evaluations per model.

Predictive performance was quantified by averaging accuracy measures across all folds and repetitions using:

• Mean absolute error (MAE), defined as the mean absolute difference between observed and predicted values;
• Root mean square error (RMSE), representing the average magnitude of the prediction error and calculated as the square root of the mean squared differences between observed and predicted values; and
• predictive R^2^ was calculated as the squared Pearson correlation coefficient between observed and predicted values obtained from cross-validation.

While predictive R^2^ represents a relative measure of predictive fit, MAE and RMSE provide absolute measures of prediction error, with MAE being less sensitive to outliers than RMSE. Lower MAE and RMSE values and higher R² values indicate better predictive performance.

To contextualize the predictive gain achieved by including biological and management predictors, baseline models consisting of an intercept and a farm-level random effect only were evaluated for the final selected dataset × lactation × trait combination (Supplementary Table S3).

The model with the lowest average RMSE was selected as the best-performing model for each trait. MAE and predictive R² were reported as complementary descriptive measures to assess robustness of prediction errors and relative predictive power.

Statistical significance of explanatory variables in the final models was assessed using Wald Chi-square tests implemented in the “car” package (version 3.0-6; Fox and Weisberg, 2019).

For MaxLN model effects are presented as multiplicative effects on the expected value of MaxLN, expressed as incidence rate ratios (IRRs) with 95% confidence intervals.

These analyses were considered exploratory and descriptive and were not used for model selection.

Model fit of the final models was assessed using marginal (R²m) and conditional (R²c) coefficients of determination following Nakagawa and Schielzeth (2013), calculated using the “MuMIn” package (version 1.47.5; Bartoń, 2023). R²m represents variance explained by fixed effects, while R²c includes both fixed and farm-level random effects; for generalized linear mixed-effects models, these should be interpreted as approximate measures of explained variance.

In addition, intraclass correlation coefficients (ICCs) were calculated from the variance components of the best-performing mixed-effects models fitted using the “lme4” package (version 1.1-35.1; Bates et al., 2015). For linear mixed-effects models, the ICC was defined as the proportion of total variance attributable to the farm-level random intercept and was calculated as σ²_farm / (σ²_farm + σ²_residual). ICCs were not reported for the Poisson generalized linear mixed-effects model (MaxLN), as variance decomposition on the observed scale is not well defined for count data.

Statistical significance was set at P < 0.05 in all analyses.

## 3 Results

### 3.1 Descriptive results

The largest validated dataset comprised 10,031 cows from 384 Swiss dairy farms, with an average herd size of 32.1 ± 17.4 cows (± standard deviation). Descriptive statistics for key traits at herd-level are presented in Table 3. As dataset_ins_ and dataset_LCP_ represent filtered subsets of this dataset, herd-level descriptive statistics were highly comparable across datasets and are therefore not shown separately.

Cows reached an average length of productive lifespan (LPL) of 1,689 ± 776 days and achieved 4.73 ± 2.00 lactations on average (MaxLN). Mean lifetime milk production (LTP) was 33,870 ± 17,068 kg ECM, corresponding to an average daily milk yield during length of productive lifespan (DMY_LPL) of 19.9 ± 3.7 kg ECM.

**Fig. 2:**
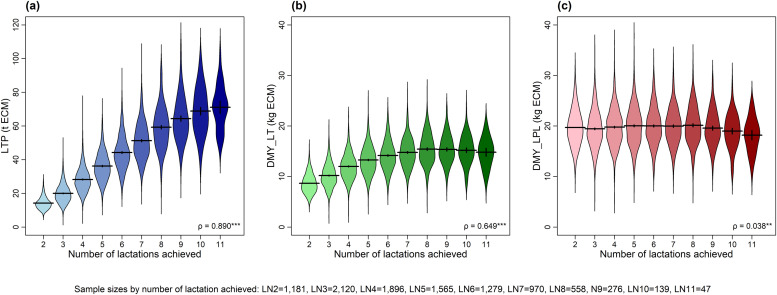
Relationship between longevity in number of lactations achieved and (a) lifetime milk production (LTP, tons of energy-corrected milk) in blue, (b) average daily milk yield during total life (DMY_LT, kg energy-corrected milk) including days from birth until culling in green, and (c) average daily milk yield during length of productive lifespan (DMY_LPL, kg energy-corrected milk) including days from first calving until culling in red.

Lifetime milk production constantly increased with every additional lactation (Fig. 2a), reflected by a strong positive correlation between number of lactations and LTP (kg ECM) (rho = 0.890). The increase averaged around 5,800 kg from second to third lactation and was highest between the third and fourth lactation (+8,231 kg increase on average). Between the fifth and eighth lactation, gains ranged between 7,000 and 8,000 kg per lactation, declining to ∼5,000 kg (eighth to ninth), < 4,500 kg (ninth to tenth), and < 2,300 kg thereafter. These values should be interpreted with caution for higher lactations, as the number of observations decreases substantially beyond the eighth lactation.

Footnote: The shape of each bean plot represents the distribution of observations, with horizontal lines indicating the average values for the respective number of lactations achieved, and vertical lines representing confidence intervals. Spearman's rank correlation coefficients (ρ) and corresponding P-values are: (a) ρ = 0.890, ^⁎⁎⁎^P < 0.0001, (b) ρ = 0.649, ^⁎⁎⁎^P < 0.0001, and (c) ρ = 0.038, ^⁎⁎^P = 0.00014. The total sample size of 10,031 cows, distributed by lactation number (LN) achieved, is provided below the figures, as it is consistent across (a), (b) and (c).

Average daily milk yield during total life (LTP divided by total lifespan, kg ECM; Fig. 2b) also increased by 1.5 kg (second to third lactation), 1.8 kg (third to fourth lactation), 1.2 kg (fourth to fifth lactation), 0.9 kg (fifth to sixth lactation), and 0.6 kg (sixth to seventh and seventh to eighth lactation) (rho = 0.648). No further increase occurred after the eighth lactation; declines of 0.2 kg (ninth to tenth lactation) and 0.4 kg (tenth to eleventh lactation) followed.

In contrast, DMY_LPL (kg ECM) showed a weak association with number of lactations achieved (rho = 0.038; Fig. 2c), remaining relatively constant up to the eighth lactation with a marginal peak in the 5th lactation. Thereafter, it declined by 0.5, 0.6 and 0.8 kg ECM with successive lactations.

### 3.2 Prediction of target traits by different datasets and first versus second lactation information

Model performance metrics obtained after 10-fold cross-validation with 5 repetitions (farm-level folds) in different datasets based on first versus second lactation information are presented in Table 5. For reference, baseline predictive performance of null mixed-effects models—including only an intercept and a farm-level random effect—evaluated for each final selected model per trait is shown in Supplementary Table S3.

**Table 5:** Model performance to predict length of productive lifespan in days (LPL), number of lactations until culling (MaxLN), lifetime milk production in kg energy-corrected milk (LTP, kg ECM), average daily milk production during productive lifespan in kg energy-corrected milk (DMY_LPL, kg ECM) using first versus second lactation information in different datasets obtaind using 10-fold cross validation (CV) with 5 repeats.

Trait	Data information		Model^3^	Model performance traits^4^		
	Dataset^1^	Lactation^2^		MAE	RMSE	Predictive R^2^ of CV
				Mean ± SD [95% CI]	Mean ± SD [95% CI]	Mean ± SD [95% CI]
LPL (days)	big	LN1	M1	650 ± 6 [648; 651]	789 ± 7 [787; 791]	0.3 ± 0.1 [0.2; 0.3]
		LN2	M1 + CI	643 ± 6 [641; 664]	782 ± 6 [781; 784]	1.3 ± 0.3 [1.2; 1.3]
	ins	LN1	M2	648 ± 8 [646; 650]	788 ± 8 [785; 790]	0.2 ± 0.2 [0.2; 0.3]
		**LN2**	**M2 + CI**	**635 ± 9 [632; 638]**	**774 ± 8 [771; 777]**	**0.7 ± 0.4 [0.6; 0.8]**
	LCP	LN1	M3	662 ± 12 [659; 666]	804 ± 14 [800; 808]	0.2 ± 0.1 [0.1; 0.2]
		LN2	M3 + CI	652 ± 11 [649; 655]	793 ± 12 [790; 797]	1.1 ± 0.4 [1.0; 1.2]
MaxLN	big	LN1	M1	3.18 ± 0.03 [3.17; 3.19]	3.75 ± 0.02 [3.74; 3.76]	0.9 ± 0.3 [0.8; 1.0]
		LN2	M1 + CI	3.18 ± 0.03 [3.17; 3.19]	3.74 ± 0.02 [3.74; 3.75]	2.4 ± 0.5 [2.2; 2.5]
	ins	**LN1**	**M2**	**3.14 ± 0.03 [3.13; 3.15]**	**3.70 ± 0.03 [3.69; 3.71]**	**0.8 ± 0.4 [0.7; 0.9]**
		LN2	M2 + CI	3.18 ± 0.04 [3.17; 3.19]	3.72 ± 0.04 [3.71; 3.73]	3.7 ± 0.7 [3.5; 3.9]
	LCP	LN1	M3	3.21 ± 0.04 [3.20; 3.22]	3.78 ± 0.03 [3.77; 3.79]	0.5 ± 0.3 [0.4; 0.5]
		LN2	M3 + CI	3.25 ± 0.03 [3.24; 3.26]	3.8 ± 0.03 [3.81; 3.82]	2.2 ± 0.5 [2.0; 2.3].
LTP (kg ECM)	big	LN1	M1	13,714 ± 158 [13,671; 13,758]	16,861 ± 176 [16,812; 16,910]	4.1 ± 0.8 [3.9; 4.3]
		LN2	M1 + CI	13,365 ± 153 [13,323; 13,407]	16,502 ± 181 [16,452; 16,552]	7.7 ± 1.0 [7.5; 8.0]
	ins	LN1	M2	13,438 ± 141 [13,399; 13,477]	16,582 ± 170 [16,534; 16,629]	4.3 ± 0.9 [4.0; 4.6]
		**LN2**	**M2 + CI**	**12,910 ± 157 [12,866; 12,953]**	**15,998 ± 159 [15,954; 16,040]**	**10.0 ± 1.1 [9.7; 10.3]**
	LCP	LN1	M3	14,050 ± 288 [13,971; 14,130]	17,232 ± 328 [17,141; 17,323]	4.2 ± 1.0 [3.9; 4.5]
		LN2	M3 + CI	13,609 ± 273 [13,533; 13,684]	16,750 ± 353 [16,652; 16,848]	7.9 ± 1.4 [7.5; 8.3]
DMY_LPL (kg ECM)	big	LN1	M1	2.22 ± 0.10 [2.19; 2.25]	2.83 ± 0.12 [2.80; 2.87]	43.3 ± 0.45 [42.0; 44.5]
		LN2	M1 + CI	2.07 ± 0.09 [2.05; 2,10]	2.65 ± 0.12 [2.61; 2.70]	50.7 ± 0.40 [49.5; 51.8]
	ins	LN1	M2	2.08 ± 0.09 [2.05; 2.10]	2.68 ± 0.12 [2.65; 2.72]	46.6 ± 0.30 [45.9; 47.4]
		LN2	M2 + CI	1.96 ± 0.12 [1.93; 1.99]	2.53 ± 0.15 [2.49; 2.57]	51.9 ± 0.37 [50.9; 52.9]
	LCP	LN1	M3	2.06 ± 0.26 [1.99; 2.14]	2.64 ± 0.33 [2.55; 2.74]	43.8 ± 0.10 [41.1; 46.6]
		**LN2**	**M3 + CI**	**1.73 ± 0.07 [1.71; 1.75]**	**2.20 ± 0.09 [2.20; 2.25]**	**64.9 ± 0.30 [64.1; 65.7]**

1 Dataset: **big** = quality-filtered dataset (10,031 cows from 384 farms); **ins** = subset of *big* including cows with complete information on number of inseminations (6,011 cows in first lactation (LN1) and 5,662 cows in second lactation (LN2) from 372 farms); **LCP** = subset of *big* including cows with validated lactation curve parameters (6,048 cows in LN1 and 6,735 cows in LN2, from 384 farms).

2 Lactation: **LN1** = first lactation; **LN2** = second lactation.

3 Models **M1- M3** were fitted as linear mixed-effects models with farm as random effect for LPL, LTP and DMY_LPL, and as generalized linear mixed-effects models with farm as random effect and a Poisson distribution for MaxLN: $M1\left(\mathrm{datase}t_{\mathrm{big}}\right):Y_{big}=\mu +AFC+nHerd+Breed+Year+se+Alp+Pers+FPR+SCC100+MY+\left(1|Farm\right)+e_{big}$ $M2\left(\mathrm{datase}t_{\mathrm{ins}}\right):Y_{ins}=\mu +AFC+nHerd+Breed+Year+se+Alp+Pers+FPR+SCC100+MY+INSEM+\left(1|Farm\right)+e_{ins,}$ $M3\left(\mathrm{datase}t_{\mathrm{LCP}}\right):Y_{LCP}=\mu +AFC+nHerd+Breed+Year+se+Alp+FPR+SCC100+MaxDMY+MaxDIM+Slope_{100-250}+\left(1|Farm\right)+e_{LCP,}$ ***Y*** = response variable; **μ** = overall mean; **AFC** = age at first calving (months); **nHerd** = herd size; **Breed** = Original Braunvieh, Brown Swiss, Simmental, Swiss Fleckvieh, Montbéliarde, Jersey, and Holstein; **Year** = year of calving (2006–2012); **se** = calving season (winter or summer); **Alp** = alpine pasturing (yes/no); **Pers** = lactation persistency (cumulative milk yield (kg) from days in milk (DIM) 101–200 divided by cumulative milk yield (kg) from DIM 1–100.); **FPR** = fat-to-protein ratio (fat content (%) / protein content (%)); **SCC100** = proportion of test-day records with somatic cell count > 100,000 cells/mL; **MY** = lactation milk yield (kg energy-corrected milk); **INSEM** = number of inseminations; **MaxDMY** = maximum daily milk yield of the lactation curve; **MaxDIM** = day in milk at peak yield; **Slope_100–250_** slope of milk yield between DIM 100 and 250; ***e*** = residual error. Calving interval (**CI**, days) was included in second-lactation models. Baseline model performance of models rated best is shown in Supplementary Table S3.

4 Model performance: Predictive performance was assessed by repeated farm-wise cross-validation (10 folds, 5 repetitons). Performance metrics were mean absolute error (**MAE**), root mean square error (**RMSE**), and predictive **R^2^ of cross validation (CV, %)**, defined as the squared Pearson correlation between observed and predicted values in test data *100, a measure for preditcitve association. Values are reported as mean ± standard deviation (SD) with 95% confidence intervals shown in brackets across all folds and repetitions. Lower MAE and RMSE and higher predictive R² indicate better predictive performance**. Final models (bold) were selected based on the lowest mean RMSE.** Baseline (null) model results are reported separately in Supplementary Table S3.

For all traits except MaxLN, models based on second-lactation (LN2) information outperformed first-lactation (LN1) models. The best-performing models for LPL and LTP were based on dataset_ins_ (LN2) including calving interval, yielding mean predictive performances of 0.7% and 10.0%, respectively. For MaxLN, predictive performance was highest when using dataset_ins_ (LN1), with a mean predictive R^2^ of 0.8%.

In contrast, prediction for DMY_LPL was strongest for the LCP-based model (M3) on second lactation data including calving interval, which achieved the lowest prediction error and the highest predictive performance (predictive R² = 64.9%).

### 3.3 Farm-level variance and intraclass correlation

Variance components from the final selected mixed-effects models are reported in Supplementary Table S4. Between-farm variability accounted for only a small proportion of total variance in LPL and LTP, whereas a substantial share of the variance in DMY_LPL was attributable to farm-level differences.

For MaxLN, a farm-level random-intercept variance component was estimated, indicating the presence of between-farm heterogeneity in this trait. However, intraclass correlation coefficients (ICCs) were not calculated due to the Poisson distribution.

### 3.4 Associations between explanatory variables and target traits in the final selected models

An overview of statistical significance of fixed effects and overall model fit for each best-performing model is presented in Supplementary Table S5. Table 6 reports estimated fixed-effect coefficients for LPL, LTP, and DMY_LPL, and multiplicative effects for MaxLN expressed as incidence rate ratios (IRRs) with 95% confidence intervals.

**Table 6:** Estimated fixed-effect regression coefficients (Est.) and standard error (SE) for explanatory traits retained in the final models selected by cross-validation for length of productive lifespan in days (LPL), number of lactations during a cow's productive lifespan (MaxLN), lifetime milk production in kg energy-corrected milk (LTP, kg ECM), and daily milk yield during length of productive lifespan (DMY_LPL, kg ECM).

		LPL (days)			Max			LTP (kg ECM)			DMY_LPL (kg ECM)		
Coefficient^1^	Contrast	Est.	SE	P	IRR	95% CI	P	Est.	SE	P	Est.	SE	P
Intercept		1,757	30	< 0.001	4.56	[4.23, 4.91]	< 0.001	34,280	593	< 0.001	19.8	0.11	< 0.001
SCC100		-95	10	< 0.001	0.98	[0.96, 0.99]	< 0.001	-1,820	214	< 0.001	-0.01	0.02	0.662
AFC		-41	11	< 0.001	0.97	[0.96, 0.99]	< 0.001	-960	233	< 0.001	0.04	0.03	0.193
MY		84	13	< 0.001	0.97	[0.96, 0.99]	< 0.001	5,611	262	< 0.001	–	–	–
Pers		34	11	0.001	1.01	[1.00, 1.02]	0.236	906	222	< 0.001	–	–	–
FPR		-35	11	0.002	1.00	[0.99, 1.01]	0.819	-785	228	< 0.001	0.29	0.03	< 0.001
CI		-3	10	0.804				-752	218	< 0.001	-0.45	0.02	< 0.001
INS		-94	11	< 0.001	0.98	[0.97, 1.00]	0.014	-3,023	231	< 0.001	–	–	–
nHerd		16	16	0.320.	1.02	[1.00, 1.03]	0.040	695	291	0.018	0.23	0.08	0.006
se_summer	Winter	-14	22	0.515	0.98	[0.96, 1.01]	0.215	-245	461	0.595	-0.02	0.05	0.760
Alp_YES	NO	102	29	< 0.001	1.02	[0.99, 1.05]	0.262	1,046	603	0.083.	-0.55	0.08	< 0.001
Breed_BS	HO	-81	40	0.045	0.95	[0.90, 0.99]	0.019	282	805	0.726	0.71	0.11	< 0.001
	OB	135	63	0.032	1.10	[1.03, 1.17]	0.006	876	1,237	0.479	-0.63	0.21	0.003
	JE	144	76	0.057	1.04	[0.96, 1.13]	0.321	0.25	1,500	1.000	0.46	0.25	0.061
	SI	20	130	0.875	1.07	[0.94, 1.21]	0.304	-1,054	2,669	0.693	-0.89	0.20	< 0.001
	SF	36	64	0.575	1.07	[1.00, 1.14]	0.058	1,277	1,300	0.326	0.22	0.12	0.073
	MO	-18	137	0.894	1.08	[0.93,1.26]	0.316	728	2,777	0.793	0.18	0.26	0.507
MaxDMY		–	–	–	–	–	–	–	–	–	3.38	0.04	< 0.001
MaxDIM		–	–	–	–	–	–	–	–	–	0.87	0.03	< 0.001
Slope_100-250_		–	–	–	–	–	–	–	–	–	1.63	0.04	< 0.001

**Notes:** Numeric predictors were standardized (mean = 0, SD = 1); estimates represent effects per one–standard deviation increase. Categorical predictors are expressed as contrasts relative to the reference level. MaxLN coefficients are reported as incidence rate ratios (IRRs) with 95% confidence intervals (95% CI), representing the multiplicative change in the expected number of lactations associated with a one standard deviation increase in the predictor, holding other variables constant. IRRs > 1 indicate higher, and IRRs < 1 lower, expected outcomes associated with a one–standard deviation increase in MaxLN.

1 **Coefficients**: SCC100 = proportion of test-day records with somatic cell count > 100,000 cells/mL; AFC = age at first calving (months); MY = lactation milk yield (kg energy-corrected milk); Pers = lactation persistency, defined as cumulative milk yield from days in milk (DIM) 101–200 divided by cumulative milk yield from DIM 1–100; FPR = fat-to-protein ratio (fat content (%) / protein content (%)); CI = calving interval (days); nHerd = herd size; INS = number of inseminations; se_summer = calving season reference set to summer, contrast = winter; Alp_YES = alpine pasturing reference set to YES; contrast = NO; Breed_BS = breed effect with Brown Swiss (BS) as reference; contrast levels: Holstein (HO), Original Braunvieh (OB), Jersey (JE), Simmental (SI), Swiss Fleckvieh (SF), Montbéliarde (MO); MaxDMY = maximum daily milk yield estimated from lactation curve models; MaxDIM = DIM at which maximum daily milk yield occurs estimated form lactation curve models; Slope_100–250_ = slope of milk yield between DIM 100 and 250 estimated from lactation curve model

Higher lactation milk yield was positively associated with higher LPL and LTP, but negatively associated with MaxLN (IRR < 1). Milk yield was represented by MaxDMY in the DMY_LPL model (results reported below).

A higher fat-to-protein ratio was associated with shorter LPL and LTP, but with higher DMY_LPL, while no significant association was observed with MaxLN. Increased lactation persistency was positively associated with LPL and LTP, but was not significantly associated with MaxLN and was replaced by Slope_100–250_ as a measure of lactation curve shape in the DMY_LPL model.

A higher number of inseminations (INS) was negatively associated with LPL, MaxLN and LTP. A longer calving interval was negatively associated with LTP and DMY_LPL but showed no significant association with LPL.

Higher SCC100 was associated with shorter LPL, lower MaxLN (IRR < 1), and lower LTP, whereas no significant association with DMY_LPL was detected. Similarly, greater age at first calving (AFC) was negatively associated with LPL, MaxLN, and LTP.

Larger herd size was associated with increased MaxLN (IRR > 1), LTP, and DMY_LPL, but not with LPL. Calving season was not significantly associated with any target traits. Alpine pasturing was associated with longer LPL and lower DMY_LPL, without significant effects on MaxLN or LTP.

Breed effects were evident for LPL, MaxLN, and DMY_LPL. Compared with Brown Swiss cows, Holstein cows showed shorter LPL and MaxLN (IRR < 1), but higher DMY_LPL, whereas Original Braunvieh cows were characterized by longer LPL and MaxLN (IRR > 1) and lower DMY_LPL.

Lactation curve parameters were exclusively used in the DMY_LPL model. Higher maximum daily milk yield, later occurrence of maximum yield, and steeper milk yield slope between days in milk 100 and 250 were all significantly associated with increased DMY_LPL.

Model explanatory power was lowest for MaxLN (R²m = 0.024; R²c = 0.043) and LPL (R²m = 0.048; R²c = 0.093), intermediate for LTP (R²m = 0.132; R²c = 0.156), and highest for DMY_LPL (R²m = 0.613; R²c = 0.726). Small differences between R²m and R²c indicate limited additional contribution of farm-level random effects (Supplementary Table S5).

## 4 Discussion

### 4.1 Implications of increasing length of productive lifespan

Dairy cattle longevity in European countries is still low, both in intensive and low-input systems (Knaus, 2009; Stiglbauer et al., 2013; Leiber et al., 2017; De Vries and Marcondes, 2020). Longevity can be considered a proxy of robustness in dairy cows because only a robust animal that is able "to maintain productivity in a wide range of environments without compromising production, health and wellbeing" (Colditz and Hine, 2016) and regularly reproduces (Friggens et al., 2017), will be able to achieve a long productive lifespan. This introduces the concept of resilience (Llonch et al., 2020), defined as the "capacity of animals to cope with short-term perturbations in their environment and return rapidly to their pre-challenge status" (Colditz and Hine, 2016). As resilience describes dynamic responses, while robustness looks at persistence of output (Llonch et al., 2020), it would be helpful for the farmer to have early detectable indicator traits (proxies) for resilience (recently investigated by Adriaens et al., 2020; Poppe et al., 2020; Ranzato et al., 2022) and robustness as criteria for phenotypic selection and replacement decisions.

Our empirical results underline the economic relevance of a long productive lifespan, which has also been reported by Dallago et al. (2021). High milk yield increases were observed between the 3rd and 6th lactation, which is in line with the results reported for Austrian organic dairy cows by Horn et al. (2012). Consistently, analysis of herdbook data from 2007–2015 of Swiss cows identified the highest maximum annual milk yield between the 5th and 7th lactation (F. Leiber and M. Holinger, Research Institute for Organic Agriculture, FiBL, Frick, Switzerland, unpublished data).

When milk yield is related to total days of life, including rearing, longer productive lifespan may provide ecological advantages due to improved resource efficiency and potentially reduced emissions per unit of product (Zehetmeyer et al., 2012). Although not investigated in the present study, it seems likely that a strong improvement of longevity would also have significant impact on methane emissions (van Middelaar et al., 2014; Grandl et al., 2016). Consistently, recent simulation studies on greenhouse gas emissions under Swiss production assumptions, even recommend the use of dual-purpose breeds as strategy for organic and mountainous dairy production to reduce the CO_2_-equivalent emission per unit of edible protein from coupled milk and meat production (Probst et al., 2019).

However, the economic optimum is influenced by various factors, such as rearing costs (Gazzarin et al., 2025), and requires further in-depth evaluation.

### 4.2 Model performance and usefulness of lactation curve parameters to predict target traits

Model performance for LPL, LTP and DMY_LPL was better using data from the second lactation compared with first-lactation data, but not for MaxLN, indicating that the additional predictive value of second-lactation data differs between target traits.

First-lactation data may have lower predictive value in most traits because cows are still growing and adapting to lactation, resulting in greater performance variability. By the second lactation, performance is typically more stable. Nevertheless, full physiological maturity in terms of milk production is usually reached only around the fifth lactation (De Vries, 2020).

Prediction for LPL, MaxLN, and LTP was strongest using dataset_ins_ (although based on different lactation information), underscoring the central role of fertility in dairy production. Fertility problems are the most frequent involuntary culling reason (Olechnowicz et al., 2016; De Vries, 2020; Schuster et al., 2020; Dallago et al., 2021), and consistently, a higher number of inseminations was negatively associated with all three traits.

For DMY_LPL, lactation curve parameters provided particularly informative predictors, although they were estimable for only 35.8% (LN1) and 47.3% (LN2) of cows. Their inclusion increased predictive R² by approximately 13 percentage points compared with the next best model based on the dataset_ins_. However, limitations of Wood’s model must be considered (Bouallegue and M’Hamdi, 2019), implying overestimation of early lactation production, underestimation of lactation peak and strong correlations among the estimated parameters (ranging between 0.70–0.90, Macciotta et al., 2005), which was also present in our data (see correlation matrix in Supplementary material Fig. S1) and can result in a high sensitivity of model estimates to data distribution (Silvestre et al., 2006). The relatively sparse Swiss test-day interval (∼34 days) likely further reduced estimation precision. Denser recording or sensor-based data might improve prediction accuracy (Adriaens et al., 2020; Poppe et al., 2020). Nevertheless, predicting length of productive lifespan and resilience ranking in first lactating cows in 27 dairy herds with automated milking systems and partly also including data from activity sensors, showed that models to estimate resilience ranking were rather suitable within, but not across herds (Adriaens et al., 2020). Another study on primiparous Dutch Holstein cows suggests that selection for low within-cow variance of milk yield as assessed by automatic milking procedures could improve longevity due to its favorable genetic correlation (Elgersma et al., 2018). On farms participating in the present study such high-density data were not available, reflecting conditions typical for many low-input systems. Thus, lactation curve parameters derived from test-day records appear useful for predicting DMY_LPL but are not helpful in predicting longevity traits.

The higher predictability of DMY_LPL aligns with the descriptive analysis showing only a weak association between DMY_LPL and number of lactations achieved (Fig. 2c). DMY_LPL remains relatively stable between the second and eighth lactation, suggesting that DMY_LPL is largely established early in life, allowing second-lactation information to provide a good approximation of lifetime-average performance.

By contrast, low predictability of longevity traits is consistent with their low heritability reported in genetic evaluations. Olechnowicz et al. (2016) reported a heritability range from 0.04 to 0.21 for longevity in their review. Comparable estimates have been reported for different populations, such as 0.15 for functional longevity in Austrian Fleckvieh cattle (Pfeiffer et al., 2015); 0.05 for days of productive life in UK Holstein cows (Pritchard et al., 2013b), and 0.09 for stayability in the herd at 60 months of age in Brazilian Holstein cows (Stefani et al., 2018). These consistently low genetic parameters underline the substantial influence of management on length of productive lifespan (e.g. Ivemeyer et al., 2008; Olechnowicz et al., 2016; Alvåsen et al., 2018; Bieber et al., 2024).

Based on the much lower reliability of models to predict LPL and MaxLN alike, which directly impact LTP, their applicability in on-farm phenotypic herd management decisions intended to improve longevity is questionable.

### 4.3 Traits useful to predict target traits

Several traits associated with the target traits in the present study have previously been linked to culling in dairy cows. Relevant associations with LPL, MaxLN, LTP, and DMY_LPL are discussed below, highlighting differences in the direction of effects and their biological interpretation across traits.

#### 4.3.1 Milk yield, fat-to-protein ratio, and lactation persistency

Milk yield showed a dual role with regard to longevity-related traits. While milk yield was positively associated with LPL and LTP, its association with MaxLN was negative. Numerous studies on culling reasons in dairy cows confirm that milk yield is relevant for longevity traits. Low milk yield is associated with an increased culling risk (e.g. Pinedo et al., 2010) and higher lifetime milk production with a lower average culling rate (Nor et al., 2014). High-producing cows are often retained longer than herdmates (e.g. Pinedo et al., 2010; Olechnowicz et al., 2016), which may explain the positive associations with LPL and LTP. However, high production levels have been reported to impair health and longevity (e.g. Ingvartsen et al., 2003; Knaus, 2009; Oltenacu and Broom, 2010; Pritchard et al., 2013a), potentially explaining the negative impact of increased lactation yield on MaxLN.

An increased fat-to-protein ratio (FPR) was associated with shorter LPL and lower LTP, supporting its role as an indicator of metabolic imbalance and elevated disease risk such as ketosis, displaced abomasum, ovarian cysts, lameness, and mastitis (Toni et al., 2011). Toni et al. (2011) reported a statistically significant higher culling hazard for cows with elevated FPR at day 7 post partum.

High FPR is induced by high lipolysis during periods of energy deficiency and serves as a ketosis indicator (FPR > 1.5) (e.g. Buttchereit et al., 2010). Its suitability as indicator for the energy status appears more pronounced during early lactation (Buttchereit et al., 2010). When relying on test-day recording, inconsistent sampling intervals may limit its usefulness for identifying high-risk cows with persistent negative energy balance (e.g. Toni et al., 2011).

Increased lactation persistency was positively associated with both LPL and LTP, indicating that cows with flatter lactation curves tend to remain productive for longer and accumulate more lifetime milk yield. However, persistency showed no association with MaxLN. In DMY_LPL models, a steeper Slope_100-250_ was positively associated with DMY_LPL.

#### 4.3.2 Fertility, alpine pasturing and longevity

A higher number of inseminations was negatively associated with LPL, MaxLN, and LTP, reflecting the reported increased culling risk for cows with fertility problems (e.g. De Vries et al., 2010; Pinedo and De Vries, 2010). Similarly, a longer calving interval was negatively associated with LTP and DMY_LPL, in line with previous findings showing increased culling risk in cows with prolonged calving intervals (Nor et al., 2014).

A genetic correlation of 0.29 was estimated between functional longevity and early fertility disorders in Austrian Fleckvieh (Pfeiffer et al., 2015). Similarly, favorable genetic correlations between productive lifespan and fertility traits (i.e. fewer inseminations, fewer days open and shorter calving intervals) were reported for Holstein Friesian cows in the UK, together with lower somatic cell count in cows with longer productive lifespan (Pritchard et al., 2013b).

Cows experiencing alpine pasturing showed longer LPL but lower DMY_LPL, indicating extended survival at the expense of daily milk output. Seasonal alpine pasturing is accompanied by prioritization of good fertility and overall health, as only fertile (pregnant) and fit cows are selected for alpine pasturing. Olmos et al. (2009) reported better reproductive performance of seasonally bred dairy cows in rotational grazing systems in Irish cows compared to their housed counterparts.

In the present dataset, 20% of the second-lactation cows experienced seasonal alpine pasturing, which was typically linked to winter calving (62.9% of second lactation cows) and thus greater fertility pressure. Such selective pressure may shorten lifespan of cows with extended calving intervals after the second parity, while potentially prolonging the lifespan of cows regularly meeting fertility benchmarks (e.g. a calving interval < 380 days). In general, alpine pasturing is considered beneficial for cattle health (Künzi et al., 1988; Ruhland et al., 1999).

#### 4.3.3 Somatic cell count and production system

Higher somatic cell count early in lactation (SCC100) was associated with shorter LPL, lower MaxLN, and reduced LTP, emphasizing the importance of udder health for survival and productivity. These findings align with reports on higher culling in Dutch dairy herds with elevated average SCC (Nor et al., 2014). Consistently, mastitis and udder problems are among the most frequent culling reasons in dairy cows (e.g. Gröhn et al., 1998; Pinedo et al., 2010). A genetic correlation of 0.63 between functional longevity and clinical mastitis was reported in Austrian Fleckvieh cattle (Pfeiffer et al., 2015), suggesting that selection for disease resistance would also improve longevity.

#### 4.3.4 Age at first calving (AFC)

Older age at first calving was negatively associated with LPL, MaxLN, and LTP, confirming AFC as an important management variable influencing lifetime performance. Numerous studies have investigated the impact of AFC on longevity-related traits and productivity.

AFC varies widely across high-producing countries ranging from 24.6 months (Netherlands) to 32.6 months (Brazil) (Dallago et al., 2021). Studies consistently show that cows calving earlier tend to stay longer in the herd and are less likely to be culled (Dallago et al., 2021).

Lower AFC was associated with increased lifetime daily milk yield and greater likelihood of a second calving, whereas late AFC was associated with increased calving interval and higher SCC in first lactation (UK Holstein cows; Eastham et al., 2018). An AFC beyond 25 months in Holstein-Friesian heifers reduced the proportion of animals reaching a third lactation (Cooke et al., 2013), and AFC above 30 months increased culling risk 1.7-fold compared with 23–24 months (Sherwin et al., 2016). Similar results were reported in Spain (Bach, 2011), Sweden (Hultgren and Svensson, 2009), and Iran (Nilforooshan and Edriss, 2004).

Overall, the evidence consistently supports beneficial effects of earlier AFC on longevity, although exceptions exist depending on herd management and regional practices (Dallago et al., 2021). However, most evidence is based on Holstein populations, limiting direct transferability to other (Swiss) breeds. In Switzerland, traditional mountain rearing systems contribute to higher average AFC in breeds such as Brown Swiss, Original Braunvieh, and Simmental.

#### 4.3.5 Breed

Dairy breeds have been selected to different degrees for milk production potential (Essl, 1998; Ingvartsen et al., 2003; Knaus, 2009), but increased milk production has been associated with negative effects on fitness and health traits (e.g. Ingvartsen et al., 2003; Oltenacu and Broom, 2010; Pritchard et al., 2013a).

In the present study, breed contributed to variation in LPL and MaxLN, with Holstein showing a negative impact on both traits. However, Holstein demonstrated higher DMY_LPL. Lower reproductive performance and survival have been reported for Dutch Holstein-Friesian with high North American gene proportions in seasonal grass-based milk production systems compared to Irish Holstein-Friesian, French Montbéliard and French Normande (Dillon et al., 2003). In organically managed Swiss sub-populations, longer LPL but lower LTP were observed in local Rhaetian Grey compared with modern Brown Swiss (Bieber et al., 2019).

## 5 Conclusion

The exploration of retrospective data confirmed the importance of length of productive lifespan for overall cow productivity. Using data from the second lactation, rather than from the first, improved model performance in predicting length of productive lifespan, lifetime milk production, and average daily milk yield over the productive lifespan. This improvement was not observed for models predicting the number of lactations achieved.

The use of lactation curve parameters derived from monthly test-day records was useful for predicting average daily milk yield during productive lifespan but cannot be recommended for predicting the other longevity-related traits.

Predictive ability differed substantially among the evaluated traits. Moderate accuracy was achieved for average daily milk yield during the productive lifetime, whereas predictions for length of productive lifespan, maximum lactation number, and lifetime milk production were markedly weaker. These findings indicate that longevity-related traits remain considerably more challenging to predict than milk production performance traits. This reduced predictability likely reflects the time-dependent nature of longevity traits, which are subject to greater variability and uncertainty over the course of an animal’s productive life.

Finally, given the limited predictability of longevity-related traits, the observed increase in milk yield up to approximately the sixth lactation highlights the importance of management and selection strategies supporting long productive lifespans and thereby enhancing the efficiency and sustainability of dairy production systems.

## Data statement

Restrictions apply to the availability of these data, which were used exclusively for this study and remain the property of Qualitas AG (Zug, Switzerland). Data may be made available upon reasonable request and subject to data use agreements with the providing organization.

## Ethical standards

‘Not applicable’.

## CRediT authorship contribution statement

**Anna Bieber:** Writing – review & editing, Writing – original draft, Visualization, Validation, Methodology, Data curation. **Dirk Hinrichs:** Writing – review & editing, Supervision, Conceptualization. **Florian N. Moser:** Writing – review & editing, Visualization, Validation, Methodology, Formal analysis, Data curation. **Ariane Maeschli:** Writing – review & editing, Validation, Project administration, Data curation. **Isabella Lora:** Writing – review & editing. **Giulio Cozzi:** Writing – review & editing, Funding acquisition, Conceptualization. **Florian Leiber:** Writing – review & editing, Supervision, Project administration, Methodology, Funding acquisition, Conceptualization.

## Declaration of competing interest

The authors declare that they have no known competing financial interests or personal relationships that could have appeared to influence the work reported in this paper.

## Appendix Supplementary materials

Image, application 1

## Footnotes

Supplementary material associated with this article can be found, in the online version, at doi:10.1016/j.vas.2026.100707.
