# Supplementary material for: Predicting longevity-related traits in Swiss low-input and organic dairy cows from herdbook data of first versus second lactation
Source: Vet Anim Sci. 2026 May 19;34:100707. doi: 10.1016/j.vas.2026.100707 (PMC13316311; doi:10.1016/j.vas.2026.100707)
Supplement: Supplementary file 1 [file mmc1.docx]

Supplementary material for the paper:

**Predicting longevity-related traits in Swiss low-input and organic dairy cows from herdbook data of first versus second lactation**

Anna Bieber^a^, Dirk Hinrichs^b^, Florian N. Moser^a^, Ariane Maeschli^a^, Isabella Lora^c^, Giulio Cozzi^c^, and Florian Leiber^a^

^a^ Department of Livestock Sciences, Research Institute of Organic Agriculture FiBL, Ackerstrasse 113, 5070 Frick, Switzerland, [anna.bieber@fibl.org](mailto:anna.bieber@fibl.org), [florian.moser@langenthal.ch](mailto:florian.moser@langenthal.ch), [ariane.maeschli@fibl.org](mailto:ariane.maeschli@fibl.org), [florian.leiber@fibl.org](mailto:florian.leiber@fibl.org)

^b^ Department of Animal Breeding, University of Kassel, Nordbahnhofstr. 1a, 37213 Witzenhausen, Germany, [dhinrichs@agrar.uni-kassel.de](mailto:dhinrichs@agrar.uni-kassel.de)

^c^ Department of Animal Medicine, Production and Health MAPS, University of Padova, Viale dell' Università 16, 35020 Legnaro (PD), Italy, [isabella.lora@unipd.it](mailto:isabella.lora@unipd.it), [giulio.cozzi@unipd.it](mailto:giulio.cozzi@unipd.it)

**Corresponding author:** Anna Bieber, Email: anna.bieber@fibl.org

Table S1 Detailed overview of validation steps and data reduction by validation step for data used in lactation curve estimation

| Step in data reduction process | Focus of validation^1^ | Reason for exclusion | First lactation data | | Second lactation data | |
| --- | --- | --- | --- | --- | --- | --- |
| Starting  dataset size | - | - | N = 9,423 cows from 384 farms | | N = 10,003 cow from 384 farms | |
|  |  |  | N | % | N | % |
| Goodness-of-fit | R^2^ ≥ 0.5 | Poor fit of lactation curve model | 883 | 9.4 | 360 | 3.6 |
| Biological plausibility of lactation curve parameters | *b* > 0 | Implausible lactation curve (linear or U-shaped) | 2,262 | - | 3,048 | - |
|  | *c* ≥ 0 |  | 633 | - | 498 | - |
|  | *b* > 0 & *c* ≥ 0 |  | 2,667 | 28.3 | 3,049 | 30.5 |
| Peak yield and peak time validation | MaxDIM ≥ 1 or ≤ 120 | Implausible peak time | 71 | 0.8 | 31 | 0.3 |
|  | MaxDMY ≥ 5 or ≤ 80 | Implausible peak yield | 153 | 1.6 | 372 | 3.7 |
| Robust outlier detection | *a* outside ± 3×IQR | Extreme values based on robust IQR criterion | 218 | - | 284 | - |
|  | *b* outside ± 3×IQR |  | 45 | - | 41 | - |
|  | *c* outside ± 3×IQR |  | 18 | - | 18 | - |
|  | MaxDMY outside ± 3×IQR |  | 382 | - | 425 | - |
|  | *a, b, c* or MaxDMY outside ± 3×IQR |  | 416 | 4.4 | 455 | 4.5 |
| Final dataset size | - | - | N = 6,048 cows (64.2%) from 384 farms | | N = 6,735 cows (67.3%) from 384 farms | |

^1^R² denotes the coefficient of determination. a, b, and c are Wood model coefficients describing the scale, ascending phase, and declining phase of the lactation curve, respectively. MaxDIM is the day in milk at peak yield, and MaxDMY is the estimated peak daily milk yield (kg) estimated by the Wood model. Robust outlier detection is based on a robust interquartile range (IQR) criterion. Within lactation quartiles Q1 (25th percentile) and Q3 (75th percentile) were calculated for each trait, with IQR = Q3 − Q1. Values < Q1 − 3×IQR or > Q3 + 3×IQR were classified as extreme values and excluded.

Table S2 Presence of different breeds by dataset

|  | Dataset^1^ and lactation number (LN)^2^, n = number of cows, (%) | | | | |
| --- | --- | --- | --- | --- | --- |
| Breed | dataset_big_ LN1 & LN2,  n = 10,031  (100%) | dataset_ins_ LN1  n = 6,011 (100%) | dataset_ins_ LN2  n = 5,662 (100%) | dataset_LCP_ LN1  n =6,048  (100%) | dataset_LCP_ LN2  n = 6,735  (100%) |
| Brown Swiss (BS) | 4,797  (47.8%) | 4,602  (76.6%) | 4,459  (78.8%) | 2,763  (45.7%) | 3,038  (45.1%) |
| Holstein (HO) | 3,075  (30.7%) | 695  (11.6%) | 556  (9.8%) | 1,919 (31.7%) | 2,170  (32.2%) |
| Jersey (JE) | 217  (2.2%) | 189  (3.1%) | 184  (3.2%) | 125  (2.0%) | 148  (2.2%) |
| Montbéliarde (MO) | 231  (2.3%) | 39  (0.6%) | 35  (0.6%) | 143  (2.4%) | 155  (2.3%) |
| Original  Braunvieh (OB) | 236  (2.4%) | 233  (3.9%) | 229  (4.0%) | 164  (2.7%) | 174  (2.6%) |
| Swiss  Fleckvieh (SF) | 1,148  (11.4%) | 199  (3.3%) | 163  (2.9%) | 717  (11.9%) | 817  (12.1%) |
| Simmental (SI) | 327  (3.2%) | 54  (0.9%) | 36  (0.6%) | 217  (3.6%) | 233  (3.5%) |

^1^Dataset: dataset**_big_** = quality-filtered dataset (10,031 cows from 384 farms); dataset_ins_ = subset of dataset**_big_** including cows with complete information on number of inseminations (6,011 cows in first lactation (LN1) and 5,662 cows in second lactation (LN2) from 372 farms); dataset**_LCP_** = subset of dataset**_big_** including cows with validated lactation curve parameters (6,048 cows in LN1 and 6,735 cows in LN2, from 384 farms).

^2^Lactation number: LN1 = first lactation, LN2 = second lactation

Table S3 Baseline predictive performance of null mixed-effects models (intercept + farm random effect) corresponding to the best-performing full models identified in Table 5 of the main manuscript, evaluated using repeated farm-wise 10-fold cross-validation (5 repeats) for length of productive lifespan (LPL, days), number of lactations until culling (MaxLN), lifetime milk production (LTP, kg ECM), and average daily milk production during productive lifespan (DMY_LPL, kg ECM)

| Trait | Data information | | |  | Model performance traits | |
| --- | --- | --- | --- | --- | --- | --- |
|  | Dataset | Lactation | Model |  | MAE  Mean ± SD [95% CI] | RMSE  Mean ± SD  [95% CI] |
|  |  |  |  |  |  |  |
| LPL  (days) | ins | LN2 | M2 + CI |  | 636 ± 4  [635; 638] | 772 ± 3  [771; 773] |
| MaxLN | ins | LN1 | M2 |  | 3.14 ± 0.03  [3.13; 3.15] | 3.71 ± 3  [3.70; 3.72] |
| LTP  (kg ECM) | ins | LN2 | M2 + CI |  | 13,530 ± 92  [13,505; 13,556] | 16,751 ± 95 [16,726; 16,778] |
| DMY_LPL (kg ECM) | LCP | LN2 | M3 + CI |  | 3.00 ± 0.08  [2.97; 3.02] | 3.80 ± 0.12 [3.76; 3.83] |

For each trait, the dataset, lactation group, and model label correspond exactly to the best-performing full model reported in Table 5. The **ins** dataset includes cows with complete insemination records (6,011 cows in first lactation (LN1) and 5,662 cows in second lactation (LN2) from 372 farms) whereas the **LCP** dataset includes cows with validated lactation curve parameters (6,735 cows in LN2, from 384 farms); M2 and M3 denote the final selected mixed-effects model structures as defined in Table 5. Although null models achieved lower MAE (mean absolute error) and RMSE (root mean squared error) under farm-wise cross-validation, they primarily reflect between-farm heterogeneity rather than predictive contributions of biological or management-related covariates.MAE and RMSE are reported as mean ± standard deviation with 95% confidence intervals. Predictive R² was not extractable for null models and is therefore not reported.

Table S4 Variance components of final mixed-effects models. Farm-level (random intercept), residual variance components and intraclass correlation coefficents (ICC) extracted from the final selected mixed-effects models for length of productive lifespan (LPL, days), maximum number of lactations (MaxLN), lifetime milk production (LTP, kg ECM), and average daily milk yield during the productive lifespan (DMY_LPL, kg ECM)

| Trait | Farm variance | Residual variance | ICC (farm) |
| --- | --- | --- | --- |
| LPL(days) | 2.69 × 10⁴ | 5.39 × 10⁵ | 0.048 |
| MaxLN | 0.004 | NA | NA |
| LTP (kg ECM) | 6.79 × 10⁶ | 2.36 × 10⁸ | 0.028 |
| DMY_LPL (kg ECM) | 1.31 | 3.17 | 0.292 |

Farm variance represents variability attributable to differences between farms (random farm intercept). Residual variance represents within-farm variability and unexplained individual variation. ICC (farm) represents the proportion of total variance attributable to between-farm differences. For the Poisson mixed-effects model (MaxLN), residual variance and ICC are not directly estimable on the response scale and are therefore not reported.

**Table S5** Type II Wald Chi-square tests for fixed effects and model performance metrics of the final mixed-effects models for target traits

| Target trait | Type II Wald Chi-square tests^2^ | | |  | | Model performance^3^ | |
| --- | --- | --- | --- | --- | --- | --- | --- |
|  | Explanatory trait^1^ | Chi-square | P-value | |  | R^2^m | R^2^c |
| Productive lifespan **(LPL, days)** | SCC100 | 84.5 | < 0.001 | |  | **0.048** | **0.093** |
|  | INS | 72.3 | < 0.001 | |  |  |  |
|  | MY | 42.8 | < 0.001 | |  |  |  |
|  | Year | 19.0 | < 0.001 | |  |  |  |
|  | Breed | 14.0 | 0.030 | |  |  |  |
|  | AFC | 12.7 | < 0.001 | |  |  |  |
|  | Alp | 11.8 | < 0.001 | |  |  |  |
|  | Pers | 10.3 | 0.001 | |  |  |  |
|  | FPR | 10.0 | 0.002 | |  |  |  |
|  | nHerd | 0.99 | 0.319 | |  |  |  |
|  | se | 0.42 | 0.515 | |  |  |  |
|  | CI | 0.06 | 0.804 | |  |  |  |
| Number of lactations reached until culling **(MaxLN)** | Breed | 21.1 | 0.002 | |  | **0.024** | **0.043** |
|  | AFC | 17.0 | < 0.001 | |  |  |  |
|  | SCC100 | 15.5 | < 0.001 | |  |  |  |
|  | MY | 14.5 | < 0.001 | |  |  |  |
|  | Year | 8.7 | 0.192 | |  |  |  |
|  | INS | 6.0 | 0.014 | |  |  |  |
|  | nHerd | 4.2 | 0.040 | |  |  |  |
|  | se | 1.5 | 0.215 | |  |  |  |
|  | Alp | 1.3 | 0.262 | |  |  |  |
|  | Pers | 1.4 | 0.236 | |  |  |  |
|  | FPR | 0.05 | 0.819 | |  |  |  |
| Lifetime milk production in kg energy corrected milk **(LTP, kg ECM)** | MY | 458.8 | < 0.001 | |  | **0.132** | **0.156** |
|  | INS | 171.7 | < 0.001 | |  |  |  |
|  | SCC100 | 72.2 | < 0.001 | |  |  |  |
|  | AFC | 16.9 | < 0.001 | |  |  |  |
|  | Pers | 16.6 | < 0.001 | |  |  |  |
|  | CI | 11.9 | < 0.001 | |  |  |  |
|  | FPR | 11.8 | < 0.001 | |  |  |  |
|  | Year | 10.9 | 0.027 | |  |  |  |
|  | nHerd | 5.7 | 0.017 | |  |  |  |
|  | Alp | 3.0 | 0.083 | |  |  |  |
|  | Breed | 1.7 | 0.944 | |  |  |  |
|  | se | 0.28 | 0.595 | |  |  |  |
| Average daily milk yield during length of productive lifespan (**DMY_LPL,  kg ECM)** | MaxDMY | 6,141.2 | < 0.001 | |  | **0.613** | **0.726** |
|  | Slope_100-250_ | 2,007.0 | < 0.001 | |  |  |  |
|  | MaxDIM | 1,186.8 | < 0.001 | |  |  |  |
|  | CI | 356.4 | < 0.001 | |  |  |  |
|  | FPR | 131.3 | < 0.001 | |  |  |  |
|  | Breed | 107.8 | < 0.001 | |  |  |  |
|  | Alp | 49.7 | < 0.001 | |  |  |  |
|  | Year | 13.2 | 0.010 | |  |  |  |
|  | nHerd | 7.7 | 0.006 | |  |  |  |
|  | AFC | 1.7 | 0.193 | |  |  |  |
|  | SCC100 | 0.19 | 0.662 | |  |  |  |
|  | se | 0.09 | 0.760 | |  |  |  |

^1^Explanatory trait: AFC = age at first calving (months); Alp = alpine pasturing (yes/no); Breed = breed effect with seven levels: Original Braunvieh (OB), Brown Swiss (BS), Simmental (SI), Swiss Fleckvieh (SF), Montbéliarde (MO), Jersey (JE), and Holstein (HO); CI = calving interval (days); FPR = fat-to-protein ratio (= fat content (%) / protein content (%)); MaxDIM = day in milk at which maximal daily milk yield is reached, estimated by the lactation curve model based on test-day records; MaxDMY = maximal daily milk yield estimated by the lactation curve model based on test-day records; MY = milk yield (kg energy-corrected milk); nHerd = herd size; Pers = lactation persistency, i.e. cumulative milk yield (kg) from days in milk (DIM) 101–200 divided by cumulative milk yield (kg) from DIM 1–100.; SCC100 = proportion of test-day records with somatic cell count > 100,000 cells/mL; Slope_100–250_ = slope of milk yield between days in milk 100 and 250 estimated by the lactation curve model based on test-day records; Year = year of calving (2006–2012)

^2^Type II Wald Chi-square tests evaluate the significance of each fixed effect in the mixed-effects models by comparing the full model with a reduced model excluding the respective effect, while retaining all other fixed effects. Reported chi-square values correspond to Wald test statistics.

^3^Marginal (R²m) and conditional (R²c) coefficients of determination were calculated following Nakagawa & Schielzeth (2013) and represent the variance explained by fixed effects alone (R²m) and by both fixed and random effects (R²c). These model-level statistics are reported once per target trait.

Figure S1 Correlation matrix of lactation curve parameters derived from the Wood model for datasets from first (LN1) and second (LN2) lactation


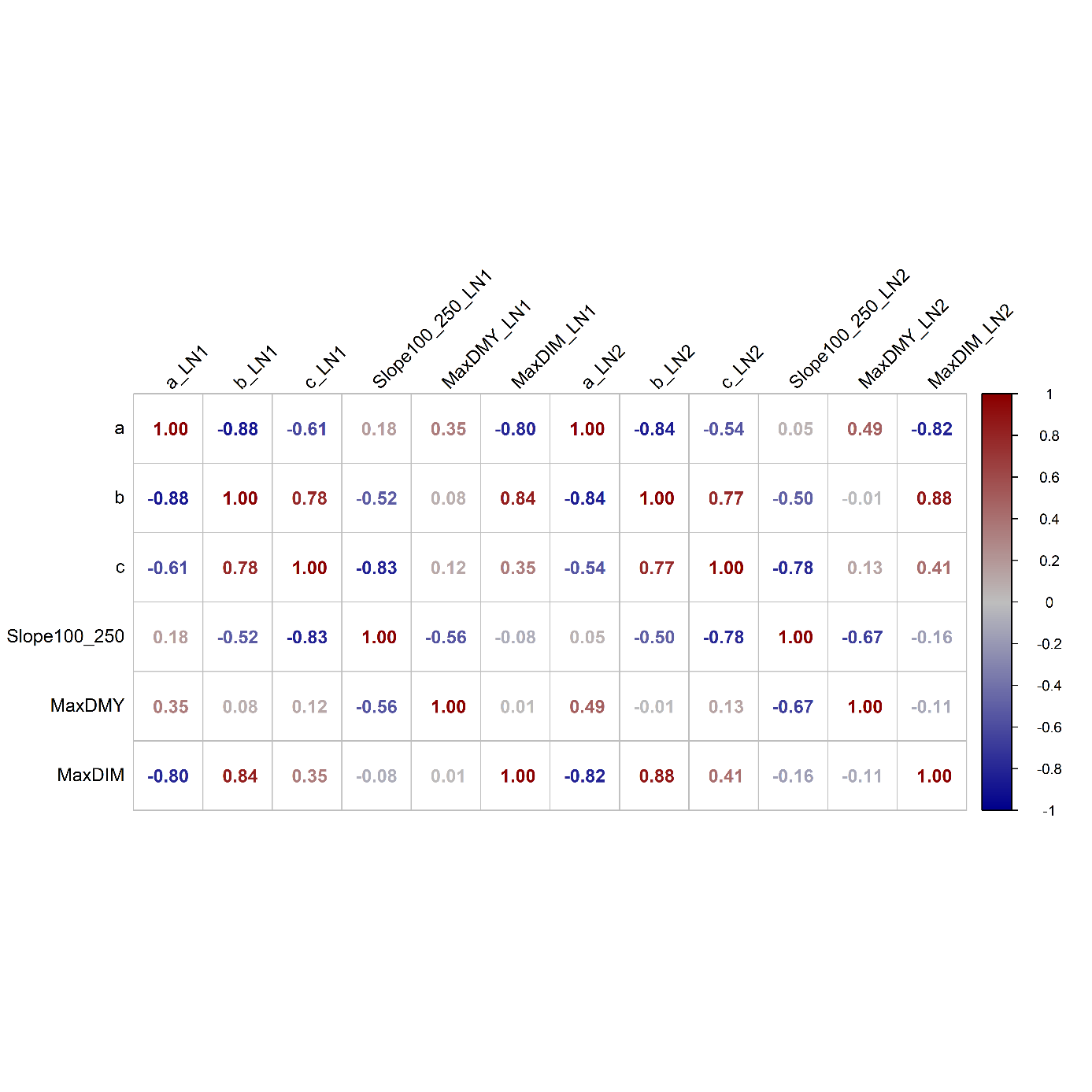


The correlation matrix illustrates the relationships between lactation curve parameters derived from the Wood model for two datasets, LN1 (first lactation, n = 6,048 cows) and LN2 (second lactation, n = 6,735 cows). The Wood model uses the parameters a, b, and c to estimate lactation curve traits. Parameter **a** represents the scale of milk yield, **b** the rate of increase in early lactation, and **c** the rate of decline after peak.

The lactation curve traits include the slope of milk yield between days in milk 100 and 250 (Slope100_250), the maximum daily milk yield (MaxDMY, kg), and the day in milk with maximum daily milk yield (MaxDIM).

Positive correlations are shown in blue, while negative correlations are displayed in red, with colour intensity indicating the strength of the correlation. Columns labelled _LN1 and _LN2 distinguish the two datasets. Rows are used in common for both datasets.
